# Alteration in the number of neuronal and non-neuronal cells in mouse models of obesity

**DOI:** 10.1093/braincomms/fcad059

**Published:** 2023-03-21

**Authors:** Mayara M Andrade, Caroline Fernandes, Leticia Forny-Germano, Rafaella A Gonçalves, Michelle Gomes, Emily Castro-Fonseca, Angela M Ramos-Lobo, Fernanda Tovar-Moll, Carlos Humberto Andrade-Moraes, Jose Donato, Fernanda G De Felice

**Affiliations:** Institute of Medical Biochemistry Leopoldo de Meis, Federal University of Rio de Janeiro, Rio de Janeiro 21941-902, Brazil; Institute of Medical Biochemistry Leopoldo de Meis, Federal University of Rio de Janeiro, Rio de Janeiro 21941-902, Brazil; Institute of Medical Biochemistry Leopoldo de Meis, Federal University of Rio de Janeiro, Rio de Janeiro 21941-902, Brazil; Centre for Neuroscience Studies, Departments of Biomedical and Molecular Sciences and Psychiatry, Queen’s University, Kingston, ON K7L 3N6, Canada; Institute of Biomedical Sciences, Federal University of Rio de Janeiro, Rio de Janeiro 21941-902, Brazil; Institute of Biomedical Sciences, Federal University of Rio de Janeiro, Rio de Janeiro 21941-902, Brazil; D’Or Institute for Research and Education, Neuroscience Laboratory, Rio de Janeiro 22281-100, Brazil; Department of Physiology and Biophysics, Institute of Biomedical Sciences, University of São Paulo, São Paulo 05508-000, Brazil; D’Or Institute for Research and Education, Neuroscience Laboratory, Rio de Janeiro 22281-100, Brazil; Institute of Medical Sciences, Federal University of Rio de Janeiro, Macaé 27930-560, Brazil; Department of Physiology and Biophysics, Institute of Biomedical Sciences, University of São Paulo, São Paulo 05508-000, Brazil; Institute of Medical Biochemistry Leopoldo de Meis, Federal University of Rio de Janeiro, Rio de Janeiro 21941-902, Brazil; Centre for Neuroscience Studies, Departments of Biomedical and Molecular Sciences and Psychiatry, Queen’s University, Kingston, ON K7L 3N6, Canada; D’Or Institute for Research and Education, Neuroscience Laboratory, Rio de Janeiro 22281-100, Brazil

**Keywords:** isotropic fractionator, obesity, neurodegeneration, neuroinflammation, neuronal loss

## Abstract

Obesity is defined as abnormal or excessive fat accumulation that may impair health and is a risk factor for developing other diseases, such as type 2 diabetes and cardiovascular disorder. Obesity is also associated with structural and functional alterations in the brain, and this condition has been shown to increase the risk of Alzheimer’s disease. However, while obesity has been associated with neurodegenerative processes, its impact on brain cell composition remains to be determined. In the current study, we used the isotropic fractionator method to determine the absolute composition of neuronal and non-neuronal cells in different brain regions of the genetic mouse models of obesity *Lep^ob/ob^* and *LepR^Null/Null^*. Our results show that 10- to 12-month-old female *Lep^ob/ob^* and *LepR^Null/Null^* mice have reduced neuronal number and density in the hippocampus compared to C57BL/6 wild-type mice. Furthermore, *LepR^Null/Null^* mice have increased density of non-neuronal cells, mainly glial cells, in the hippocampus, frontal cortex and hypothalamus compared to wild-type or *Lep^ob/ob^* mice, indicating enhanced inflammatory responses in different brain regions of the *LepR^Null/Null^* model. Collectively, our findings suggest that obesity might cause changes in brain cell composition that are associated with neurodegenerative and inflammatory processes in different brain regions of female mice.

## Teaser

This study identifies neuronal loss in the hippocampus and frontal cortex of two mouse models of obesity (*Lep^ob/ob^* and *LepR^Null/Null^*) using the isotropic fractionator. *LepR^Null/Null^* displayed increased non-neuronal cells, primarily glial cells, in the hippocampus, frontal cortex and hypothalamus. Quantitative measures showed that *LepR^Null/Null^* exhibits aggravated neurodegeneration when compared to *Lep^ob/ob^*.

## Graphical Abstract

**Graphical Abstract:**
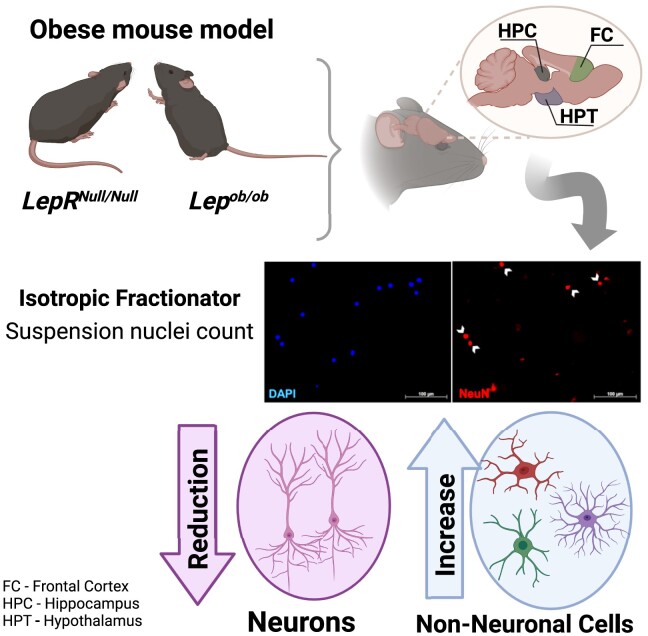

## Introduction

Obesity is a multifactorial chronic disease defined by excessive fat accumulation that might impair health, and it is classically diagnosed as having a body mass index (BMI) superior to 30 kg/m^2^ (World Health Organization). Unhealthy diets, sedentarism, susceptible metabolic profiles and vulnerable social and socioeconomic status are important contributors to obesity.^1,2^ Moreover, obesity was suggested to decrease life expectancy by up to 20 years,^3,4^ and according to the World Health Organization, 650 million people aged 18 or over were obese in 2016 and this number is expected to triplicate by 2050.^5,6^

Obesity is associated with a higher risk of developing several disorders, including type 2 diabetes (T2D),^7^ cardiovascular diseases^8^ and dementia.^9,10^ Neurodegeneration is being increasingly associated with obesity, as obese individuals are at a higher risk of developing cognitive impairment,^11^ and atrophy of brain regions,^12^ including the frontal cortex^13^ and hippocampus.^14^ Neurodegenerative processes and cognitive impairment were also reported in mouse models of obesity.^15,16^ Nonetheless, the association between obesity and alterations in brain cell numbers is still unclear.

Brain size and function can be affected by many factors, including the absolute number and density of neuronal and glial cells. Among the approaches used to determine brain cell composition, the isotropic fractionator (IF)^17,18^ was introduced by Herculano-Houzel and Lent^19^ in 2005 as an innovative, simple and accurate method as an alternative to more time-consuming stereological techniques.^19-21^ The IF method involves transforming highly anisotropic brain structures into homogeneous, isotropic suspensions of cell nuclei, which can be identified immunocytochemically as neuronal or non-neuronal nuclei and then counted. In the cerebral cortex of humans, 70% of the non-neuronal population identified using the IF method is composed of glial cells (astrocytes, microglia and oligodendrocytes).^22-24^ The IF technique has been used in animal models of Alzheimer’s disease and central nervous system (CNS) disorders.^25,26^ In the current study, we use the IF technique to determine the absolute brain cell composition of rodent models of obesity.

The *Lep^ob/ob^* is a monogenic mouse model of obesity widely used in the study of metabolic disorders.^27,28^ This model holds spontaneous mutations in the obese (*ob*) gene that encodes leptin, rendering mice unable to produce this hormone.^29-31^ The *LepR^Null/Null^* mouse model presents a transcriptional blocker in the leptin receptor (*LepR*) gene that alters the leptin pathway, causing a deficit in the leptin receptor.^32,33^ Both *Lep^ob/ob^* and *LepR^Null/Null^* mice exhibit accelerated weight gain with triplicated body size at the adult age, hyperphagia, transient hyperglycaemia and reduced energy expenditure.^29,34,35^ Cognitive decline, synaptic loss, impaired neurogenesis and neuroinflammation were also reported in these models of obesity.^15,36,37^ Of relevance, similar mutations in the *ob* and *LepR* genes were found in obese humans.^38-40^

In the current study, we investigated the precise brain cell composition of female *Lep^ob/ob^* and *LepR^Null/Null^* mice. We quantified neuronal and non-neuronal cells in the hippocampus, frontal cortex and hypothalamus of *Lep^ob/ob^*, *LepR^Null/Null^* and wild-type (WT) mice. We demonstrate decreased neuronal and increased non-neuronal cell numbers and densities in the hippocampus of obese mice. Furthermore, we observed an increase in non-neuronal cells in the frontal cortex and hypothalamus of *LepR^Null/Null^* mice. Our results suggest an association between neurodegenerative and neuroinflammatory processes in the hippocampus, frontal cortex and hypothalamus that may be associated with cognitive impairment observed in obese rodent models and humans.

## Materials and methods

### Animals

Ten-month-old females *Lep^ob/ob^* (The Jackson Laboratory; stock #000632) and 12-month-old females *LepR^Null/Null^* mice (The Jackson Laboratory; stock #018989) were produced in the University of São Paulo. *Lep^ob/ob^* mice were generated by crossing heterozygous B6.Cg-*Lep^ob^*/J mice. The brains of males and females are substantially different^41,42^ and cannot be directly compared. Therefore, considering the poor availability of information about female mouse brains in the literature, the current study focuses on female mice. Moreover, due to the high mortality of *LepR^Null/Null^* mice after 9 months of age, the sample size for this experimental group is smaller than for the other groups in the current study.

Homozygotes were used as the spontaneous model of obesity, and 10- to 12-month-old WT mice (C57Bl/6J) littermate were used as WT controls. All mice were housed in climate-controlled rooms on a 12-h light–dark cycle, with standard rodent chow and water access *ad libitum*. All procedures were conducted in accordance with the Guiding Principles for the Care and Use of Research Animals. Animals were euthanized in compliance with the recommendations of the Animal Care Committee for the Federal University of Rio de Janeiro (Rio de Janeiro, Brazil) Process number: 01200.001568/2013-87.

### Perfusion and tissue preparation

In a room designated for animal experimentation inside the laboratory, mice were anaesthetized with a single intraperitoneal injection of a ketamine (20 mg/kg) and xylazine (2 mg/kg) mix. We checked constantly the level of anaesthesia when mice were unconscious and did not move in response to noxious and external stimuli, including pain. If mice appeared to be responding to touch or awakening, a reinjection with 50% of the initial dose of ketamine was applied. Subsequently, mice were perfused transcardially, inside a fume hood, using a peristaltic metering pump (Milan #BP600/2) with 150 mL of 0,9% saline solution, followed by 100 mL of 4% paraformaldehyde.

Brains were post-fixed overnight in 4% paraformaldehyde at 4°C and stored in phosphate buffer solution (PBS) 0.1 M until dissection of the regions of interest (ROIs). Area delimitations were selected according to the ROIs below: frontal cortex: 90° from the corpus callosum genus forward and upward; hippocampus: after localizing the optic chiasma (OC) with the brain in a ventral position, all the encapsulated region beneath corpus callosum was obtained; hypothalamus: we inserted a clamp curve in the middle of the OC and a cut was made with 1 mL deep until backwards to the mammillary body. Immediately after dissection, the frontal cortex, hippocampus and hypothalamus were weighed and stored in PBS solution to be subsequently subjected to the IF. The PBS solution was weekly replaced by a new one until samples were processed.

### Isotropic fractionator

IF was performed as previously described.^19^ This technique transforms highly anisotropic tissues into a suspension of intact nuclei.^43^ Briefly, post-fixed ROIs were chemomechanically disrupted in a homogenizer containing dissociation solution (buffer-detergent solution with 40 mM sodium citrate and 1% Triton™ X-100) until all fragments were dissociated. The duration of the homogenization process ranged from 2.5 to 4 min. Cells in suspension were transferred to a falcon tube, and the homogenizer was rinsed a few times with 0.1 M PBS to collect any residual cells and prevent nuclei loss.

### Quantification of suspension nuclei

**Figure 1:**
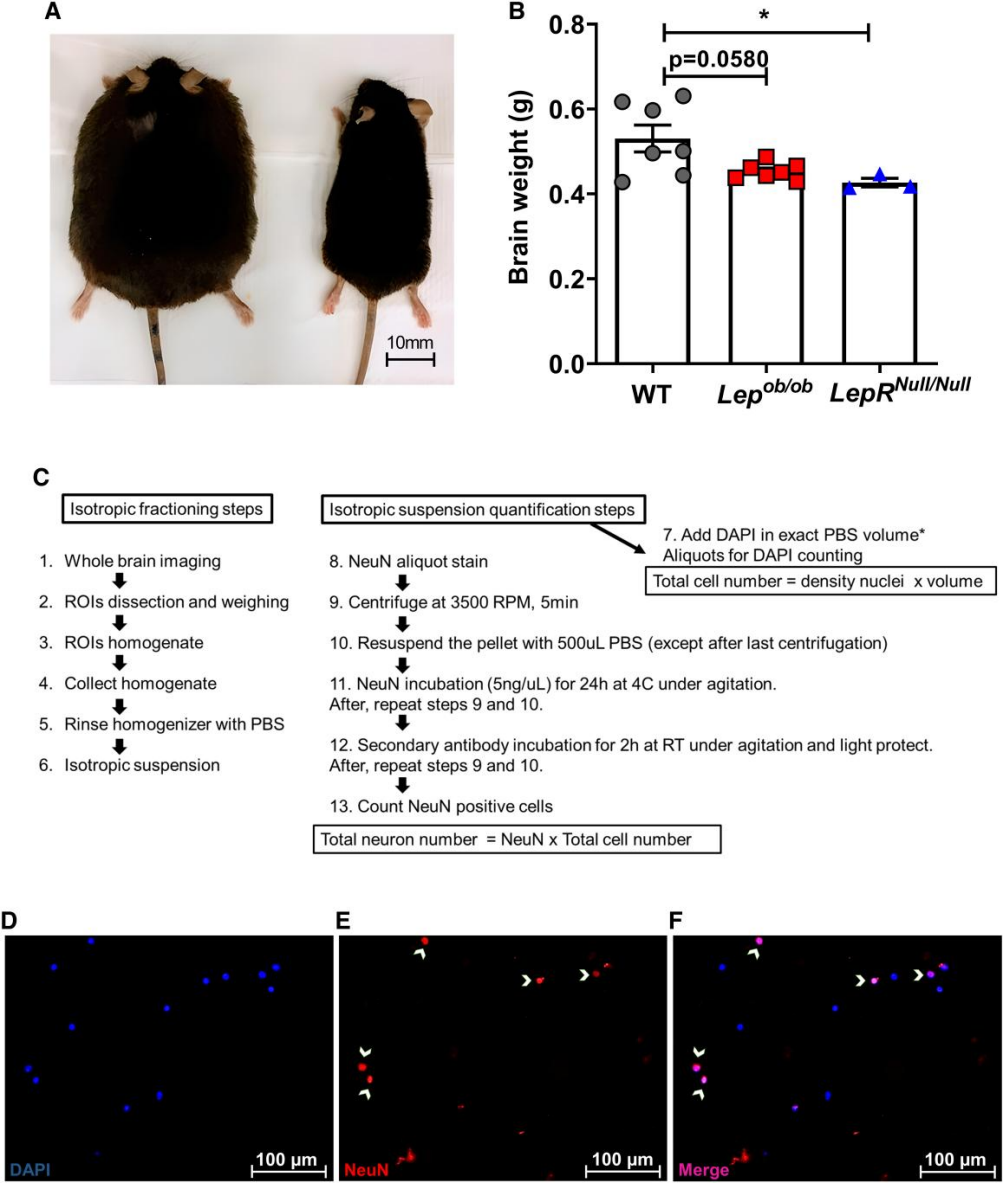
**Isotropic fractionator (IF) technique workflow.** (**A**) Representative image of a monogenic mouse model of obesity (10–12 months) and a WT mice (10 months) showing differences in body size. (**B**) Brain weight of *Lep^ob/ob^* (*n* = 7; *P* = 0.058) and *LepR^Null/Null^* (*n* = 3; *P* = 0.0465) obese mice when compared to WT mice (*n* = 7). (**C**) Workflow diagram illustrating the IF steps that involve the transformation of highly anisotropic brain structures into isotropic suspensions of cell nuclei, allowing for rapid quantitative analysis of cell composition of different brain regions. The isotropic suspension volume (*V*_F_) obtained for each brain region analysed was for hypothalamus—*V*_F_ = 2.5 mL; hippocampus—*V*_F_ = 5.0 mL; and frontal cortex—*V*_F_ = 5.0 mL. (**D**–**F**) Representative images of the hippocampus nuclei suspension after fractionation, with staining and immunocytochemistry to DAPI (**D**), NeuN (**E**) and merge between DAPI and NeuN (**F**) and the white arrowheads indicate nuclei doubly stained with DAPI and NeuN. Scale bars: 100 µm. The dot and bar graph were expressed as mean ± SEM; one-way ANOVA with Tukey’s *post hoc* test. Significant differences were indicated by *P < 0.05.

To quantify the absolute cell number, an aliquot of the isotropic suspension was collected after previous homogenization of the entire fraction at least 30 times. A solution with 2% 4′,6-diamidino-2-phenylindole (DAPI, 20 mg/L D9542; Sigma) was then added to each aliquot, and samples were smoothly homogenized to disperse the nuclei present in the suspension. For the quantification of DAPI stained nuclei, at least four 10 μL aliquots of the suspension were collected and applied in two hemocytometers (Neubauer chamber), then analysed under a fluorescence microscope (Zeiss Axio Imager) with a ×20 objective. It is important to emphasize that the suspension was considered homogeneous only if the coefficient of variation (CV) calculated for the four analysed aliquots was ≤15%. Otherwise, the count was repeated with new aliquots until CV ≤ 15%. The number of nuclei (*N*_N_) present in the aliquot was obtained from the average values acquired in DAPI’s quantification (Fig. 1D). The total amount of nuclei was obtained by multiplying the total suspension volume (*V*_F_) and the nuclei density, which was determined by multiplying the average number of nuclei (*N*_N_) by the dilution factor. This dilution factor is a factor associated with the total number of squares considered for counting, whose value is 15 625.^19,43^

The neuronal number was calculated using an additional aliquot of each ROI collected after previous homogenization. First, the aliquots were centrifuged at 6000 rpm for 5 min, and the pellet was washed three times with PBS 0.1 M (6000 rpm for 5 min in each washing step). The final pellet was then incubated with 2 µL of BSA 5%, 30 µL of normal donkey serum (NDS), 1 µL of primary antibody anti-NeuN (1:200; Millipore; #MAB377) and 167 µL of PBS 0.1 M for 24 h under agitation at 4°C. Following incubation with primary antibody, the aliquots were washed as described above, and the pellet was incubated with 2 µL of BSA 5%, 30 µL of NDS, 10 µL of DAPI (20 mg/L; Thermo Fisher Scientific; #62248), 1 µL of secondary antibody Alexa Fluor® 594 (1:500; Invitrogen; #A-21203) and 460 µL of PBS 0.1 M for 2 h at room temperature (RT) under agitation. To prepare samples for analyses, the aliquots were centrifuged and rinsed twice in PBS 0.1 M, and the pellet was resuspended in PBS 0.1 M. The volume of PBS 0.1 M was 100 µL for the hypothalamus and 200 µL for the hippocampus and frontal cortex. The absolute number of neurons was calculated by multiplying the proportion of NeuN^+^ nuclei and the total cell number (Fig. 1C). The neuronal density was obtained by dividing the number of neurons by the weight of the brain region. The absolute number of non-neuronal cells (oligodendrocytes + astrocytes + microglia + endothelial and ependymal cells) was obtained by subtracting the absolute number of neurons from the total cell number, and non-neuronal density was calculated considering the regional weight.

### Statistical analysis

All groups were homogeneously separated taking into account genotyping, age and weight. For quantification of suspension nuclei, all samples were randomly allocated to avoid bias and were quantified by more than one experimenter who did not know their identification. All results were analysed using GraphPad Prism 8 (GraphPad Software, Inc.). Data sets were assessed for normality with Shapiro–Wilk test, and statistical significance was assigned when *P* < 0.05. Graphs are expressed as mean ± SEM, and statistical analyses were performed using one-way ANOVA followed by the Tukey *post hoc* test. For statistical significance between groups, values were included as indicated, i.e. *P < 0.05, **P < 0.01, ***P < 0.001 and ****P < 0.0001. Pearson correlation was performed with a 95% confidence interval and two-tailed parameters.

## Results

### *LepR^Null/Null^* mice showed an increase in the absolute number and density of total cells in the hypothalamus

To evaluate whether obesity is associated with morphological alterations in the brain, we initially analysed the total brain weight of obese mice. Ten- to 12-month-old female *Lep^ob/ob^* and *LepR^Null/Null^* mice showed an accentuated increase in body weight when compared to WT group (Fig. 1A), which is in agreement with the literature.^31,44-46^*Lep^ob/ob^* and *LepR^Null/Null^* mice had a 14.49% (*P* = 0.058) and a 19.57% (*P* = 0.046) reduction in total brain weight, respectively, when compared to WT (Fig. 1B).

**Figure 2:**
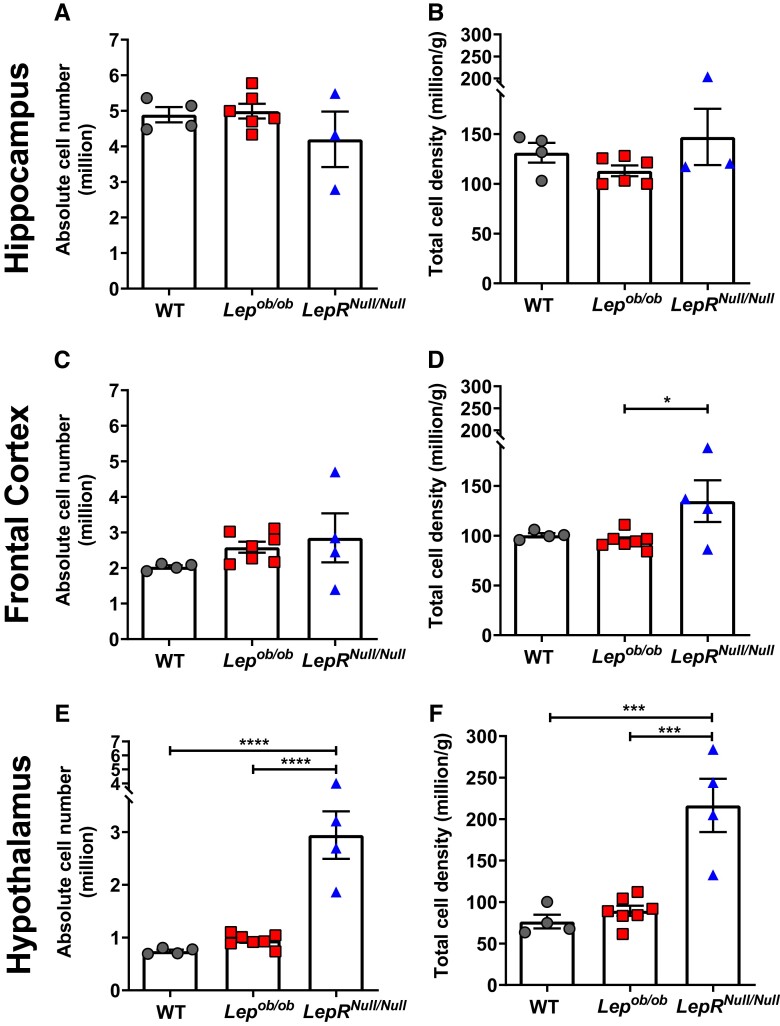
**Changes in total cell number and density, especially in *LepR^Null/Null^*.** (**A** and **B**) Absolute total cell number (**A**) and cell density (**B**) in the hippocampus of WT (*n* = 4). *Lep^ob/ob^* (*n* = 6) and *LepR^Null/Null^* mice (*n* = 3). (**C** and **D**) Absolute total cell number (**C**) and cell density (**D**) in the frontal cortex of WT (*n* = 4), *Lep^ob/ob^* (*n* = 7) and *LepR^Null/Null^* mice (*n* = 4). (**E** and **F**) Absolute total cell number (**E**) and cell density (**F**) in the hypothalamus of WT (*n* = 4), *Lep^ob/ob^* (*n* = 7) and *LepR^Null/Null^* mice (*n* = 4). Data are shown as mean ± SEM; symbols represent individual animals; *P*-values were calculated from one-way ANOVA followed by Tukey’s *post hoc* test. Significant differences were indicated by *P < 0.05, ***P < 0.001 and ****P < 0.0001.

We next determined the total cell number in the hippocampus, frontal cortex and hypothalamus of *Lep^ob/ob^* and *LepR^Null/Null^* mice compared to WT. Each ROI was dissected, weighed and processed for the IF analyses according to the steps described in the IF workflow (Fig. 1C). We did not find significant differences in the absolute cell number (Fig. 2A) and total cell density (Fig. 2B) in the hippocampus when comparing *Lep^ob/ob^*, *LepR^Null/Null^* and WT mice. In the frontal cortex, while the total cell number did not differ between the groups (Fig. 2C), the total cell density showed a 41.68% increase in *LepR^Null/Null^* compared to *Lep^ob/ob^* mice (*P* = 0.034) but did not differ significantly from WT mice (Fig. 2D). In the hypothalamus, although no differences were found between *Lep^ob/ob^* and WT mice, *LepR^Null/Null^* mice exhibited a 295.73% increase (*P* < 0.0001) in total cell number (Fig. 2E) and a 183.05% increase (*P* < 0.001) in cell density (Fig. 2F) compared to WT mice, and a 210.99% increase (*P* < 0.0001) in total cell number and a 142.15% increase (*P* < 0.001) in cell density when compared to *Lep^ob/ob^* mice (Fig. 2E and F).

Collectively, our findings indicate that obesity did not alter the total cell number and density in the hippocampus and frontal cortex of both mouse models compared to WT mice and that *LepR^Null/Null^* mice exhibit greater total cell number and density than *Lep^ob/ob^* and WT mice, particularly in the hypothalamus.

### *Lep^ob/ob^* and *LepR^Null/Null^* mice showed a reduction in the absolute number of neurons in the hippocampus

In addition to the analyses of total cell number and density, the IF method allows researchers to quantify specific cellular populations in the brain. Therefore, we next aimed to evaluate possible changes in the number of neuronal and non-neuronal cells in *Lep^ob/ob^* and *LepR^Null/Null^* compared to WT mice. We first quantified neuronal nuclei in the cell suspension obtained from the IF method (Fig. 1C) by analysing cells immunoreactive for the neuronal nuclear marker, NeuN, in specific brain regions.

**Figure 3:**
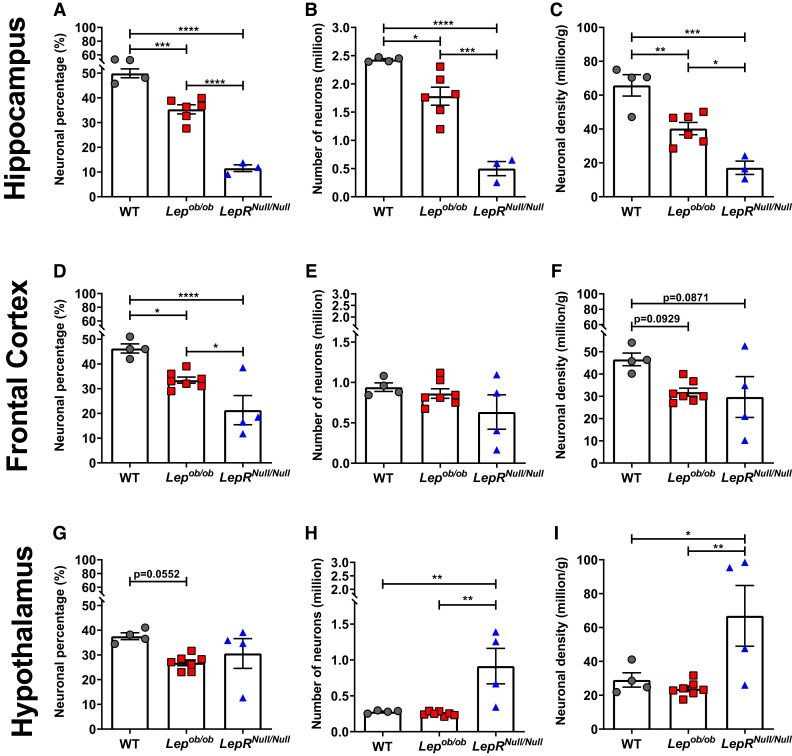
**Neuronal changes in the brain of obese mouse models.** (**A**–**C**) Neuronal population in the hippocampus including percentage (**A**), absolute number (**B**) and density (**C**) of cells in WT (*n* = 4), *Lep^ob/ob^* (*n* = 6) and *LepR^Null/Null^* mice (*n* = 3). (**D**–**I**) Neuronal population in the frontal cortex and hypothalamus, including percentage (**D** and **G**, respectively), number (**E** and **H**, respectively) and density (**F** and **G**, respectively) of cells in WT (*n* = 4), *Lep^ob/ob^* (*n* = 7) and *LepR^Null/Null^* mice (*n* = 4). Data are shown as mean ± SEM; symbols represented individual animals; *P*-values were calculated from one-way ANOVA followed by Tukey’s *post hoc* test. Significant differences were indicated by *P < 0.05, **P < 0.01, ***P < 0.001 and ****P < 0.0001.

In the hippocampus, we found that obese mice had a significant reduction in the percentage of neuronal cells (Fig. 3A), particularly *LepR^Null/Null^* mice (*P* < 0.0001). We detected a 26.64% reduction (*P* = 0.0166) in the number (Fig. 3B) and a 38.75% reduction (*P* = 0.0063) in the density (Fig. 3C) of neuronal cells in *Lep^ob/ob^* mice when compared to WT mice. *LepR^Null/Null^* mice showed a more severe decrease in neuronal number and density, 79.46% (*P* < 0.0001) and 73.99% (*P* = 0.0002), respectively, when compared to WT mice (Fig. 3B and C). In the frontal cortex, we observed the same pattern of reduced percentage of neuronal cells found in the hippocampus (Fig. 3D). However, the number of neurons did not differ among the groups (Fig. 3E). We also observed a trend of decrease in neuronal density in *Lep^ob/ob^* (31.63%) and *LepR^Null/Null^* (36.28%) models compared to WT controls (*Lep^ob/ob^*, *P* = 0.0923; *LepR^Null/Null^*, *P* = 0.0871) (Fig. 3F). In the hypothalamus, only *Lep^ob/ob^* mice showed a trend of decrease in the percentage of neuronal cells (Fig. 3G, *P* = 0.0552), but not neuronal number (Fig. 3H) and density (Fig. 3I) when compared to WT mice. *LepR^Null/Null^* mice exhibited a 227.71% increase (*P* = 0.009) in total neuronal number (Fig. 3H) and a 130.25% increase (*P* = 0.0353) in neuronal density (Fig. 3I) compared to WT.

Our findings show that obesity is associated with reduced absolute neuronal number and density in the hippocampus, suggesting that this condition may favour neurodegenerative processes in a brain region important for memory consolidation and cognition. Furthermore, the hypothalamus of *LepR^Null/Null^* mice had a higher number and density of neuronal cells than *Lep^ob/ob^* and WT (Fig. 3H and I), thus exhibiting the same pattern observed in total cell count (Fig. 2E and F). The results observed in the hypothalamus of *LepR^Null/Null^* mice might be due to specific genotypic characteristics of this model.

### *LepR^Null/Null^* mice showed an increased density of non-neuronal cells in the hippocampus, frontal cortex and hypothalamus

Chronic and exacerbated inflammatory responses have been reported in the obese brain.^34,47^ Therefore, we next aimed to determine whether the number and density of non-neuronal cells, mainly composed of glia,^22,23^ were altered in obese mice. The absolute number of non-neuronal cells was obtained by subtracting the total number of neurons from the total number of cells in each brain ROI of *Lep^ob/ob^*, *LepR^Null/Null^* and WT mice.

**Figure 4:**
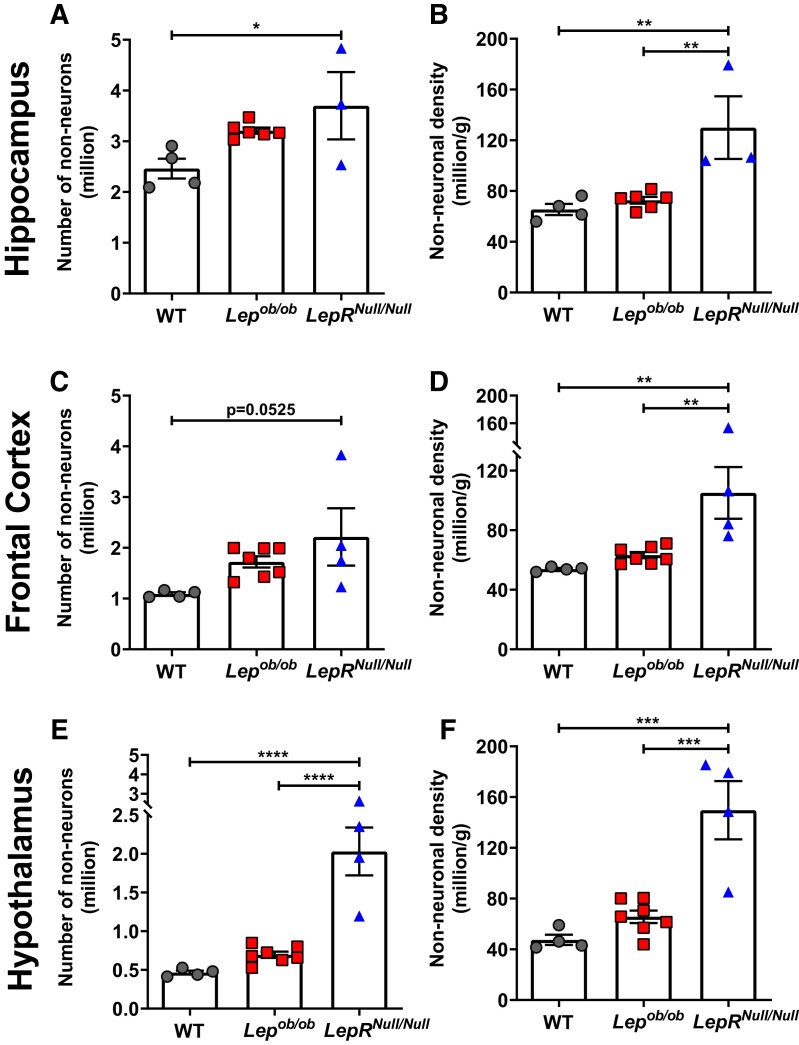
**Increased non-neuronal cell number and density in different brain regions of *LepR^Null/Null^* mice.** (**A** and **B**) Absolute number (**A**) and density (**B**) of non-neuronal cells in the hippocampus of WT (*n* = 3), *Lep^ob/ob^* (*n* = 6) and *LepR^Null/Null^* mice (*n* = 3). (**C** and **D**) Absolute number (**C**) and density (**D**) of non-neuronal cells in the frontal cortex of WT (*n* = 4), *Lep^ob/ob^* (*n* = 7) and *LepR^Null/Null^* mice (*n* = 4). (**E** and **F**) Absolute number (**E**) and density (**F**) of non-neuronal cells in the hypothalamus of WT (*n* = 4), *Lep^ob/ob^* (*n* = 7) and *LepR^Null/Null^* mice (*n* = 4). Data are shown as mean ± SEM; symbols represented individual animals; *P*-values were calculated from one-way ANOVA followed by Tukey’s *post hoc* test. Significant differences were indicated by *P < 0.05, **P < 0.01, ***P < 0.001 and ****P < 0.0001.

In the hippocampus, we observed a 50.31% increase (*P* = 0.041) in the number of non-neuronal cells in *LepR^Null/Null^* mice compared to WT animals (Fig. 4A). These obese mice also showed a higher density of non-neuronal cells than WT (98.61%, *P* = 0.0049) or *Lep^ob/ob^* (78.73%, *P* = 0.007) mice (Fig. 4B). In the frontal cortex, the number of non-neuronal cells did not differ between the groups (Fig. 4C), but non-neuronal cell density was greater in *LepR^Null/Null^* mice compared to WT or *Lep^ob/ob^* mice (Fig. 4D). In the hypothalamus, the number and density of non-neuronal cells did not differ between *Lep^ob/ob^* and WT mice but were substantially increased in *LepR^Null/Null^* compared to WT (for number 336.40%, *P* < 0.0001; for density 215.38%, *P* = 0.0002), or *Lep^ob/ob^* mice (for number 191.44%, *P* < 0.0001; for density 127.93%, *P* = 0.0005) (Fig. 4E and F).

Overall, *LepR^Null/Null^* mice exhibited increased in non-neuronal density in all three brain regions analysed when compared to both WT and *Lep^ob/ob^* mice. Since the non-neuronal cell population is mainly composed of astrocytes, microglia and oligodendrocytes,^22-24^ our findings suggest exacerbated neuroinflammatory responses in different brain regions of *LepR^Null/Null^* mice.

### The absolute number of neuronal, non-neuronal and total cells increases with hypothalamic weight

**Figure 5:**
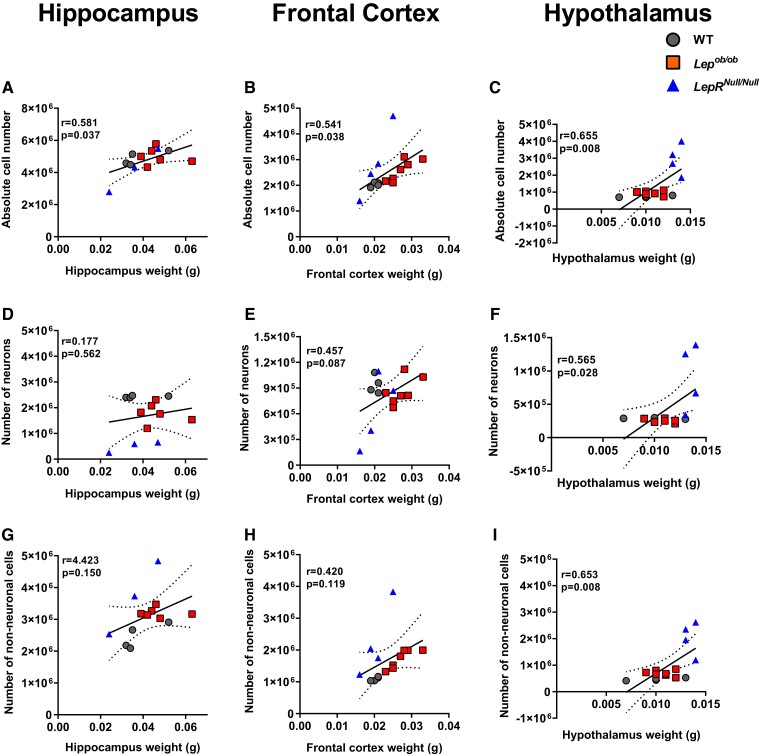
**Correlations between total cell number and regional brain weight.** (**A**–**C**) Pearson correlations between the number of total cells and weight in the hippocampus (**A**), the frontal cortex (**B**) and the hypothalamus (**C**) among WT, *Lep^ob/ob^* and *LepR^Null/Null^* mice. (**D**–**F**) Pearson correlations between neuronal number and weight in the hippocampus (**D**), frontal cortex (**E**) and hypothalamus (**F**) among WT, *Lep^ob/ob^* and *LepR^Null/Null^* mice. (**G**–**I**). Pearson correlations between the number of non-neuronal cells and weight in the hippocampus (**G**), frontal cortex (**H**) and hypothalamus (**I**) among WT, *Lep^ob/ob^* and *LepR^Null/Null^* mice. Correlation coefficients (*r*) and *P*-values are shown in the graphs.

We next investigated whether the number of neuronal, non-neuronal and total brain cells correlated with the weight of each ROI. Our results show that the total number of cells in the brains of WT, *Lep^ob/ob^* and *LepR^Null/Null^* mice positively correlate with the total weight of hippocampus (Fig. 5A; *r* = 0.501; *P* = 0.037), frontal cortex (Fig. 5B; *r* = 0.541; *P* = 0.038) and hypothalamus (Fig. 5C; *r* = 0.655; *P* = 0.008). The same pattern was observed when we correlated the number of neuronal cells with the total weight of hippocampus (Fig. 5D; *r* = 0.177; *P* = 0.562), frontal cortex (Fig. 5E; *r* = 0.457, *P* = 0.087) and hypothalamus (Fig. 5F; *r* = 0.565, *P* = 0.028). The number of non-neuronal cells did not correlate with hippocampal (Fig. 5G; *r* = 0.423; *P* = 0.150) or frontal cortex (Fig. 5H; *r* = 0.420; *P* = 0.119) weight but showed a positive correlation with hypothalamic weight (Fig. 5I; *r* = 0.653; *P* = 0.008). The hypothalamus was the brain region with the highest mass-cell number, particularly in *LepR^Null/Null^* mice. These findings suggest that the hypothalamus of the *LepR^Null/Null^* model has severe gliosis and highlight an important difference between the *Lep^ob/ob^* and *LepR^Null/Null^* obesity models.

### Non-neuron/neuron ratio is greater in *LepR^Null/Null^* mice and correlates with neuronal density in the hippocampus and frontal cortex

**Figure 6:**
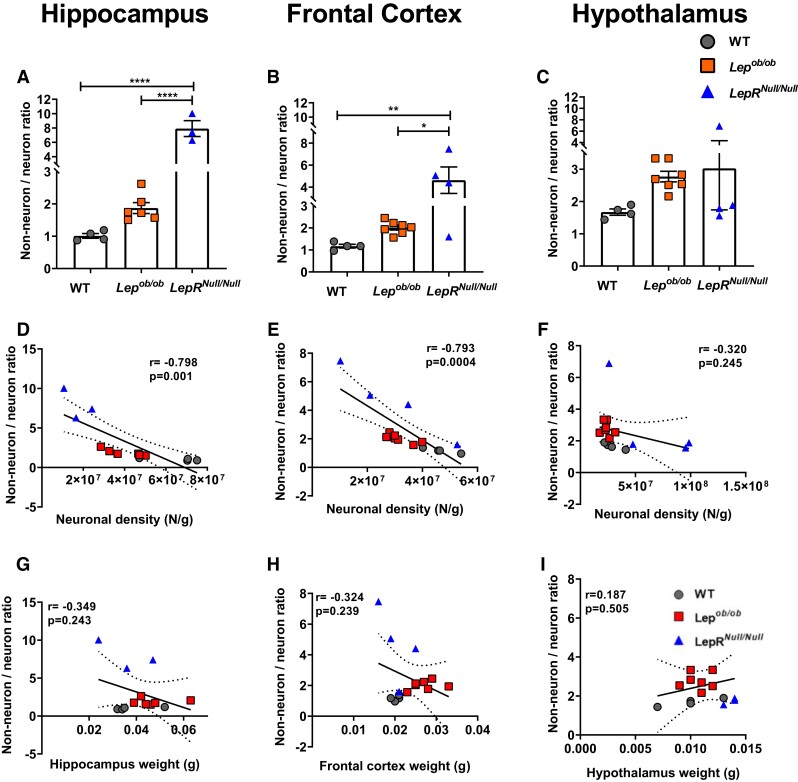
**Changes in non-neuron/neuron ratio in different brain regions of *LepR*^Null/Null^ mice.** (**A**–**C**) Non-neuron/neuron ratio in the hippocampus (**A**), frontal cortex (**B**) and hypothalamus (**C**) of WT (*n* = 4), *Lep^ob/ob^* (*n* = 6) and *LepR^Null/Null^* (*n* = 3) mice. (**D**–**F**) Pearson correlations between non-neuron/neuron ratio and neuronal density in the hippocampus (**D**), frontal cortex (**E**) and hypothalamus (**F**) of WT, *Lep^ob/ob^* and *LepR^Null/Null^* mice. (**G**–**I**) Pearson correlations between non-neuron/neuron ratio and weight in the hippocampus (**G**), frontal cortex (**H**) and hypothalamus (**I**). Data are shown as mean ± SEM; *P*-values were calculated from one-way ANOVA followed by Tukey’s *post hoc* test. Significant differences are illustrated by *P < 0.05, **P < 0.01 and ****P < 0.0001. Correlation coefficients (*r*) are shown in the graphs.

Several studies have shown that glial cells play a crucial role in brain function during physiological and pathological conditions. In neurodegenerative diseases, particularly in Alzheimer’s disease, brain inflammation has been shown to underlie neuronal dysfunction and cognitive impairment in mouse models of disease.^23,48^ We then sought to determine the ratio between non-neuronal and neuronal cells in different brain regions of WT, *Lep^ob/ob^* and *LepR^Null/Null^* mice. Our results show that *LepR^Null/Null^* mice have a greater non-neuron/neuron ratio in the hippocampus (Fig. 6A) and frontal cortex (Fig. 6B) when compared to WT and *Lep^ob/ob^* mice. No differences were observed in this ratio in the hypothalamus (Fig. 6C). We next investigated the relationship between non-neuron/neuron ratio and neuronal density, and we found a negative correlation between these variables in the hippocampus (Fig. 6D; *r* = −0.798, *P* = 0.001) and frontal cortex (Fig. 6E; *r* = −0.793, *P* = 0.0004). No statistically significant correlation was observed in the hypothalamus (Fig. 6F; *r* = −0.320, *P* = 0.245). Finally, we evaluated the correlation between the non-neuron/neuron ratio and the weight of each ROI. We found a negative correlation between these variables in the hippocampus (Fig. 6G; *r* = −0.349, *P* = 0.014), and no changes in the frontal cortex (Fig. 6H; *r* = −0.334, *P* = 0.239) and hypothalamus (Fig. 6I; *r* = 0.187, *P* = 0.505). In Table 1, we present the summary of all quantifications and statistical parameters for each ROI analysed in the current study.

**Table 1:** Cellular composition of each ROI in obese mouse models (*Lep^ob/ob^* and *LepR^Null/Null^*) and WT mice

	Hippocampus					
	WT		*Lep^ob/ob^*		*LepR^Null/Null^*	
	Estimates	CV (%)	Estimates	CV (%)	Estimates	CV (%)
ROI weight (mg)	38.25 ± 9.25	24.19%	44.30 ± 3.39	7.64%	28.88 ± 6.26	21.69%
Total cell number (millions)	4.89 ± 0.43	8.70%	4.99 ± 0.51	10.20%	4.20 ± 1.35	32.22%
Total cell density (million/g)	131.23 ± 19.84	15.12%	113.03 ± 13.38	11.84%	147.09 ± 48.95	33.28%
Percentage of NeuN^+^ nuclei	49.90 ± 0.04	7.23%	35.36 ± 0.05	12.84%	11.55 ± 0.02	20.23%
Number of neurons (millions)	2.43 ± 0.04	1.58%	1.78 ± 0.39	21.95%	0.50 ± 0.22	43.17%
Neuronal density (million/g)	65.79 ± 12.62	19.19%	40.29 ± 8.88	22.04%	17.11 ± 6.81	39.81%
Number of non-neurons (millions)	2.46 ± 0.39	15.89%	3.21 ± 0.15	4.64%	3.70 ± 1.15	31.06%
Non-neuronal density (million/g)	65.45 ± 8.76	13.38%	72.74 ± 6.49	8.92%	129.98 ± 42.80	32.93%

	Frontal cortex					
	WT		*Lep^ob/ob^*		*LepR^Null/Null^*	
	Estimates	CV (%)	Estimates	CV (%)	Estimates	CV (%)
ROI weight (mg)	20.25 ± 0.96	4.73%	27.14 ± 3.29	12.11%	20.30 ± 3.067	18.09%
Total cell number (millions)	2.03 ± 0.09	4.55%	2.58 ± 0.41	15.95%	2.85 ± 1.38	48.41%
Total cell density (million/g)	100.42 ± 4.28	4.26%	95.06 ± 8.25	8.68%	134.67 ± 41.89	31.11%
Percentage of NeuN^+^ nuclei	46.25 ± 0.04	7.97%	33.43 ± 0.03	9.90%	21.33 ± 0.12	55.23%
Number of neurons (millions)	0.94 ± 0.11	11.19%	0.86 ± 0.16	18.16%	0.63 ± 0.43	67.08%
Neuronal density (million/g)	46.56 ± 5.71	12.26%	31.83 ± 4.76	14.95%	29.67 ± 18.34	61.80%
Number of non-neurons (millions)	1.09 ± 0.07	6.01%	1.72 ± 0.29	16.94%	2.21 ± 1.13	50.99%
Non-neuronal density (million/g)	53.86 ± 1.48	2.75%	63.22 ± 5.56	8.79%	105.00 ± 34.70	33.05%

	Hypothalamus					
	WT		*Lep^ob/ob^*		*LepR^Null/Null^*	
	Estimates	CV (%)	Estimates	CV (%)	Estimates	CV (%)
ROI weight (mg)	10.08 ± 2.34	23.25%	10.71 ± 1.11	10.39%	13.60 ± 0.54	3.93%
Total cell number (millions)	0.74 ± 0.06	7.55%	0.95 ± 0.12	12.64%	2.94 ± 0.90	30.57%
Total cell density (million/g)	76.50 ± 16.46	21.52%	89.47 ± 16.27	18.19%	216.52 ± 64.41	29.75%
Percentage of NeuN^+^ nuclei	37.56 ± 0.03	7.37%	26.81 ± 0.03	11.47%	30.58 ± 0.12	39.49%
Number of neurons (millions)	0.28 ± 0.01	4.92%	0.25 ± 0.03	11.25%	0.91 ± 0.49	54.00%
Neuronal density (million/g)	29.05 ± 8.49	29.21%	23.83 ± 4.40	18.47%	66.90 ± 35.84	53.58%
Number of non-neurons (millions)	0.46 ± 0.05	10.72%	0.69 ± 0.11	15.60%	2.03 ± 0.62	30.51%
Non-neuronal density (million/g)	47.44 ± 7.99	16.85%	65.64 ± 13.05	19.87%	149.62 ± 45.88	30.66%

The values are shown as mean ± SD. CV is the coefficient of variation and was calculated as standard deviation (SD)/mean.

## Discussion

In the current study, we applied the IF technique to determine alterations in the absolute cell composition and density of the brain in two rodent models of obesity, the *Lep^ob/ob^* and the *LepR^Null/Null^* mice. We provide evidence that obesity is associated with reduced brain size and altered cell number and density in different brain regions of mice. Specifically, we found a reduced number of neurons in the hippocampus of *Lep^ob/ob^* and *LepR^Null/Null^* mice, and an elevated number of non-neuronal cells in the frontal cortex, hippocampus and hypothalamus of *LepR^Null/Null^* mice.

IF and stereology are among the techniques used in the quantitative analysis of brain cell composition.^17,18,24^ Stereology combined with histochemical and immunocytochemical staining was previously applied to quantify the number of neuronal and astrocyte populations in the striatum of post-mortem brains from obese humans, and no differences were observed when these samples were compared to a lean control group.^49^ In mice fed with a high-fat diet (HFD), stereology analyses demonstrated a significant increase in neuropeptide Y (NPY) and proopiomelanocortin (POMC) cell volumes and distribution in the hypothalamus.^50^ However, the use of IF is more advantageous than stereology, as it is faster, cheaper and easier to perform and replicate.^20,51^ Moreover, the IF method has better staining with NeuN antibodies and eliminates problems with tissue shrinkage, which leads to more reliable results when analysing neuronal populations.^51,52^ Therefore, IF has the potential to more precisely evaluate the cellular composition of specific brain regions in obese mouse models.

Our results demonstrate that *Lep^ob/ob^* and *LepR^Null/Null^* have reduced brain weight when compared to WT mice (Fig. 1B). An early study from Bereiter and Jeanrenaud,^53^ in 1978, indicated morphological changes in the brains of *Lep^ob/ob^*, including a reduction in brain weight and cortical brain volume,^53^ and *LepR^Null/Null^* mice were shown to have reduced brain weight.^32^ Reduced hippocampal volume was also recently detected in *Lep^ob/ob^* mice.^46^ Comparable results of decreased brain weight and brain volume were demonstrated in overweight and obese humans, indicating that obesity is associated with morphological changes in the brain, including in the hippocampus and frontal cortex.^54,55^ Magnetic resonance imaging (MRI) studies reported brain atrophy in adult subjects with a BMI higher than 30 and in diabetic individuals.^4,56^ A 24-year follow-up study including women over 60 years old observed that those with temporal lobe atrophy had a BMI 1.1–1.5 kg/m^2^ higher than women without brain atrophy. Specifically, the risk of atrophy was shown to increase by 13–16% per 1.0 kg/m^2^ increase in BMI.^57^

Brain atrophy can result from a reduction in neuronal and/or non-neuronal cells and may differentially impact the pathophysiology of diseases, as we see in Alzheimer’s disease and other types of dementia.^58-60^ In the current study, we found a robust and significant reduction in the percentage, number and density of neurons in the hippocampus of *Lep^ob/ob^* and *LepR^Null/Null^* mice (Fig. 3A–C). The hippocampus is an important brain region for learning and memory that is significantly affected in neurodegenerative diseases.^61^ Molecular modifications in the hippocampus, such as microglial activation, decreased neurogenesis and apoptosis, are directly related to the onset of dementia.^61,62^ Moreover, studies using diet-induced obesity models demonstrated that neuroinflammation is an important feature of obesity, including in the hippocampus,^63^ that is associated with decreased neurogenesis,^15,64^ cognitive impairment^56,65^ and activation of apoptosis.^66^ In obesity, adipocytes produce pro-inflammatory cytokines that can cross the blood–brain barrier (BBB) and induce microglial activation and neuronal damage.^34,67^ Indeed, pharmacological inhibition of microglial activation in obese mice prevented dendritic spine loss and cognitive decline,^68^ highlighting an association between neuroinflammation, impaired neurogenesis and neurodegeneration.^69^ Moreover, since neuronal loss can result from impaired neurogenesis and/or enhanced cell apoptosis,^70,71^ it is possible that both mechanisms are involved in the neuronal cell reduction we observed in obese mice.

Although we did not find differences in the number and density of neuronal cells in the frontal cortex of *LepR^Null/Null^* and *Lep^ob/ob^* mice when compared to WT, our results show increased non-neuronal density in *LepR^Null/Null^* mice (Fig. 4E). Furthermore, a similar increase was observed in the hippocampus of *LepR^Null/Null^* mice (Fig. 4D). A greater number of non-neuronal cells, which are mainly composed of glia,^22^ indicates enhanced inflammatory responses. Studies using diet-induced obesity models demonstrated a significant increase in inflammatory markers in the frontal cortex, such as tumour necrosis factor α (TNF-α) and interleukin-1 β (IL-1β),^72^ and also demonstrated that HFD-induced obesity could cause a harmful influence on brain cortex bioenergetics.^73^ Obese humans with high BMI (in particular, over 40) and without neurological diseases have reduced mRNA expression of the anti-inflammatory cytokine interleukin 10 (IL-10) and increased mRNA expression of the enzyme associated with cytotoxicity and apoptosis inducible nitric oxide synthase in the frontal cortex.^74^ These alterations, especially associated with the dysregulation of pro- or anti-inflammatory cytokines, may indicate an intensified neuroinflammatory response in this brain region.^75^

The *Lep^ob/ob^* mouse is a monogenic model of obesity widely used in the study of metabolic disorders.^15,37^*Lep^ob/ob^* mice have a single mutation in the leptin gene (*ob*) on chromosome 6 that causes a premature termination in leptin synthesis and prevents the secretion of the bioactive form of this hormone.^28^ Since leptin is a key hormone in the regulation of energy expenditure and food intake, this alteration results in the appearance of early obesity dysfunctions, such as insulin resistance associated with hyperglycaemia and hyperinsulinaemia, reduced energy expenditure and adipose tissue inflammation, but resistance to develop diabetes.^37,76^*LepR^Null/Null^* is a genetically modified model that had the insertion of a Lox-flanked transcription-blocking cassette (*loxTB*) between exons 16 and 17 of the *LepR* gene, preventing the transcription of downstream sequences, which leads to premature termination of the intracellular domain.^32,77^ Thus, *LepR^Null/Null^* mice have a phenotype similar to *Lepr^db^*, which is another transgenic mouse model of obesity lacking the long (*B*) isoform of the *LepR*.^78^ Phenotypically, *LepR^Null/Null^* and *Lepr^db^* are morbidly obese, hyperphagic and hyperglycaemic, with hyperinsulinaemia, and have reduced energy expenditure in addition to being infertile.^32,33,77^*LepR^Null/Null^* and *Lep^db^* mice are both susceptible to developing diabetes. *Lep^db^* undergoes pancreatic tissue atrophy, including loss of beta cells, which leads to reduced insulin secretion and severe hyperglycaemia combined with obesity and diabetes at a very early age.^79^*Lep^ob/ob^* and *LepR^Null/Null^* exhibit similar obese phenotypes, and herein we observed comparable reductions in brain weight. However, *LepR^Null/Null^* mice showed more significant changes in cellular number and composition compared to *Lep^ob/ob^* or WT mice, including a greater density of non-neuronal cells in all brain regions.

The *LepR^Null/Null^* model develops diabetes at a very early age.^32^ Higher blood glucose and low insulin levels are associated with oxidative stress, decreases in synaptic density and cell death.^80-82^ Therefore, although the two mouse models used in the current study had similar reductions in brain weight and similar body weights, different levels of circulating glucose and insulin may be associated with our divergent findings in brain cellular composition when comparing *Lep^ob/ob^* and *LepR^Null/Null^* mice and with the higher variability observed in the *LepR^Null/Null^* group. We also highlight that due to a significant increase in mortality of *LepR^Null/Null^* mice after 9 months of age, our study includes a small sample size for the *LepR^Null/Null^* experimental group. Future studies evaluating the relationship between glucose and insulin tolerance with neuronal loss in *Lep^ob/ob^* and *LepR^Null/Null^* mice will be important to determine the contribution of these variables to changes in brain cellular composition in obesity.

The hypothalamus is a key brain region for the control of food intake, metabolic regulation and energy homeostasis and is mainly targeted by leptin, insulin and ghrelin in the agouti-related peptide (AgRP) and POMC neurons.^83^ Hypothalamic alterations, including exacerbated inflammatory responses and injury, were reported in humans and in animal models of obesity and neurodegenerative diseases.^84^ A stereology study using young HFD mice demonstrated that an 8-week hypercaloric diet increased the volume and decreased neuronal density in the hypothalamus.^85^ We here found an increased density of total cells (183%), neuronal cells (130.25%) and non-neuronal cells (215.38%) in the hypothalamus of *LepR^Null/Null^* mice when compared to WT mice (Figs 2F, 3I and 4F). Intriguingly, we did not find differences when comparing these same parameters between *Lep^ob/ob^* and WT mice. Hypothalamic mass was also higher in *LepR^Null/Null^* than in WT or *Lep^ob/ob^* mice (Fig. 1B), and it positively correlated with total cell number, neuronal cell number and non-neuronal cell number (Fig. 5B, F and I). A distinct profile of hypothalamic alteration was thus demonstrated in the *LepR^Null/Null^* model.

We here propose that obesity is associated with alterations in brain cellular composition. Therefore, although obesity may drive these alterations in *Lep^ob/ob^* and *LepR^Null/Null^* mice, these changes may also occur prior to the onset of obesity and therefore favour an obese phenotype in these models. Future studies investigating this possibility are warranted. Nonetheless, we note that a substantial increase in body weight was observed in the first month of life in both *Lep^ob/ob^* and *LepR^Null/Null^* mice. Moreover, studies using other techniques reported neuronal loss and increased glial cells in WT mice fed with a HFD.^86-88^ Diet-induced obesity was shown to trigger early hypothalamic abnormalities in mice.^87^ After 1 day on HFD, mice displayed altered expression of proinflammatory cytokines, microglia and astrocyte markers, mitochondria and autophagy-related proteins, and hypothalamic neuropeptides. After 7 days on HFD, astrogliosis and increased BBB permeability were also observed.^87^ Stereological and histopathological examination of the brains of rodents fed with HFD found hippocampal neuronal loss, increased oxidative stress and cellular inflammatory response.^88^

The incidence of metabolic disorders, including obesity and type 2 diabetes, has significantly increased in the last decades. These diseases have a great impact on modern society and represent a major risk factor for developing neurodegenerative diseases.^89,90^ Quantitative comparisons of brain cell composition in mouse models of obesity may represent a potential mechanistic substrate for a causal link between obesity and neurodegenerative disorders. Nonetheless, future studies evaluating the brain cellular composition of younger mouse models of obesity will be important to determine the timeline of the changes observed in the current study and to uncover whether these changes occur after or prior to the onset of obesity. Analyses in non-transgenic mouse models, including animals exposed to a HFD, will be also relevant to seek this information.

## Data Availability

All the results obtained are relevant for the findings of this study and can be obtained from the corresponding authors upon request.

## Abbreviations

AgRP =: agouti-related peptide
BBB =: blood–brain barrier
BMI =: body mass index
CNS =: central nervous system
CV =: coefficient of variation
DAPI =: 4′,6-diamidino-2-phenylindole
HFD =: high-fat diet
IF =: isotropic fractionator
IL-10 =: interleukin 10
*LepR* =: leptin receptor
*N*_N_ =: number of nuclei
NPY =: neuropeptide Y
OC =: optic chiasma
POMC =: proopiomelanocortin
ROIs =: regions of interest
RT =: room temperature
T2D =: type 2 diabetes
*V*_F_ =: total suspension volume
WT =: wild-type
